# Low LDL-C and High HDL-C Levels Are Associated with Elevated Serum Transaminases amongst Adults in the United States: A Cross-sectional Study

**DOI:** 10.1371/journal.pone.0085366

**Published:** 2014-01-15

**Authors:** Zhenghui Gordon Jiang, Kenneth Mukamal, Elliot Tapper, Simon C. Robson, Yusuke Tsugawa

**Affiliations:** 1 Division of Gastroenterology and Hepatology, Department of Medicine, Beth Israel Deaconess Medical Center, Harvard Medical School, Boston, Massachusetts, United States of America; 2 Division of General Medicine and Primary Care, Department of Medicine, Beth Israel Deaconess Medical Center, Harvard Medical School, Boston, Massachusetts, United States of America; 3 Harvard University Interfaculty Initiative in Health Policy, Harvard University, Cambridge, Massachusetts, United States of America; Institute of Medical Research A Lanari-IDIM, University of Buenos Aires-National Council of Scientific and Technological Research (CONICET), Argentina

## Abstract

**Background:**

Dyslipidemia, typically recognized as high serum triglyceride, high low-density lipoprotein cholesterol (LDL-C) or low high-density lipoprotein cholesterol (HDL-C) levels, are associated with nonalcoholic fatty liver disease (NAFLD). However, low LDL-C levels could result from defects in lipoprotein metabolism or impaired liver synthetic function, and may serve as *ab initio* markers for unrecognized liver diseases. Whether such relationships exist in the general population has not been investigated. We hypothesized that despite common conception that low LDL-C is desirable, it might be associated with elevated liver enzymes due to metabolic liver diseases.

**Methods and Findings:**

We examined the associations between alanine aminotransferase (ALT), aspartate aminotransferase (AST) and major components of serum lipid profiles in a nationally representative sample of 23,073 individuals, who had no chronic viral hepatitis and were not taking lipid-lowering medications, from the National Health and Nutrition Examination Survey (NHANES) from 1999 to 2010. ALT and AST exhibited non-linear U-shaped associations with LDL-C and HDL-C, but not with triglyceride. After adjusting for potential confounders, individuals with LDL-C less than 40 and 41–70 mg/dL were associated with 4.2 (95% CI 1.5–11.7, p = 0.007) and 1.6 (95% CI 1.1–2.5, p = 0.03) times higher odds of abnormal liver enzymes respectively, when compared with those with LDL-C values 71–100 mg/dL (reference group). Surprisingly, those with HDL-C levels above 100 mg/dL was associated with 3.2 (95% CI 2.1–5.0, p<0.001) times higher odds of abnormal liver enzymes, compared with HDL-C values of 61–80 mg/dL.

**Conclusions:**

Both low LDL-C and high HDL-C, often viewed as desirable, were associated with significantly higher odds of elevated transaminases in the general U.S. adult population. Our findings underscore an underestimated biological link between lipoprotein metabolism and liver diseases, and raise a potential need for liver evaluation among over 10 million people with particularly low LDL-C or high HDL-C in the United States.

## Introduction

Measurement of triglyceride and cholesterol concentrations among different lipoproteins as part of the serum lipid panel is a routine part of cardiovascular disease risk stratification. It is rarely considered a useful screening tool for the evaluation of liver diseases, yet there is reason to think otherwise. The liver is the central hub for lipid metabolism and controls the production and clearance of serum lipoproteins [1], [2]. Hence, liver disease is likely to be intimately related to serum lipid levels.

Dyslipidemia typically refers to elevated LDL-C or triglyceride or low HDL-C, a pattern that is associated with cardiovascular risk and is also frequently seen in nonalcoholic fatty liver disease (NAFLD) [3], [4]. NAFLD, a spectrum of disease ranging from hepatic steatosis to nonalcoholic steatohepatitis (NASH) and cirrhosis, is the most common form of chronic liver disease and the most likely cause of elevated transaminases in otherwise healthy individuals [4], [5]. Up to 33–46% of the US population may have NAFLD, among whom 3% eventually develop end-stage liver disease [6]–[8]. Hepatic steatosis, the critical “first hit” of NAFLD, fundamentally results from imbalanced intrahepatic lipid homeostasis leading to triglyceride accumulation [9]. Insulin resistance, as seen in metabolic syndrome, a common cause of dyslipidemia, is thought to be a primary driver of NAFLD [6], [7], [10], [11]. In population-based epidemiological studies, factors associated with elevated ALT include higher age, male gender, high waist circumference, high triglyceride level, and biomarkers consistent with insulin resistance [4].

However, steatosis does not always concord with dyslipidemia. Two classic examples are abetalipoproteinemia and familial hypobetalipoproteinemia (FHBL), genetic conditions characterized by inadequate assembly and secretion of apolipoprotein B (apoB)-containing lipoproteins from hepatocytes [12]–[17]. Both conditions paradoxically lead to apparently desirable serum lipid profiles but significant hepatic steatosis. Discordance also occurs in cirrhosis, even early compensated or occult-cirrhosis, in which decreased liver synthetic function results in decreased apolipoprotein synthesis and lipoprotein particle secretion, resulting in low circulating LDL-C [18]. For these reasons, a serum lipid panel mistakenly considered “optimal” could represent occult liver disease. However, this association has not been carefully studied to validate its presence and prevalence.

In this context, we used data from serial iterations of the National Health and Nutrition Examination Survey (NHANES) and examined the relationship between the values of serum lipid panel and liver transaminases, a marker for chronic liver diseases among the US population.

## Methods

### Study Population

NHANES is a nationally representative cross-sectional study conducted by the National Center for Health Statistics at the Centers for Disease Control and Prevention [19], 20. Participants are selected using a stratified multistage probability design with oversampling of certain age and ethnic groups [19]. Provided sample weights allow for inferences to the civilian non-institutionalized population of the US. All participants were interviewed for demographic, socioeconomic, health and dietary information. Information on alcohol and tobacco consumption was available for participants 20 years and older.

**Figure 1:**
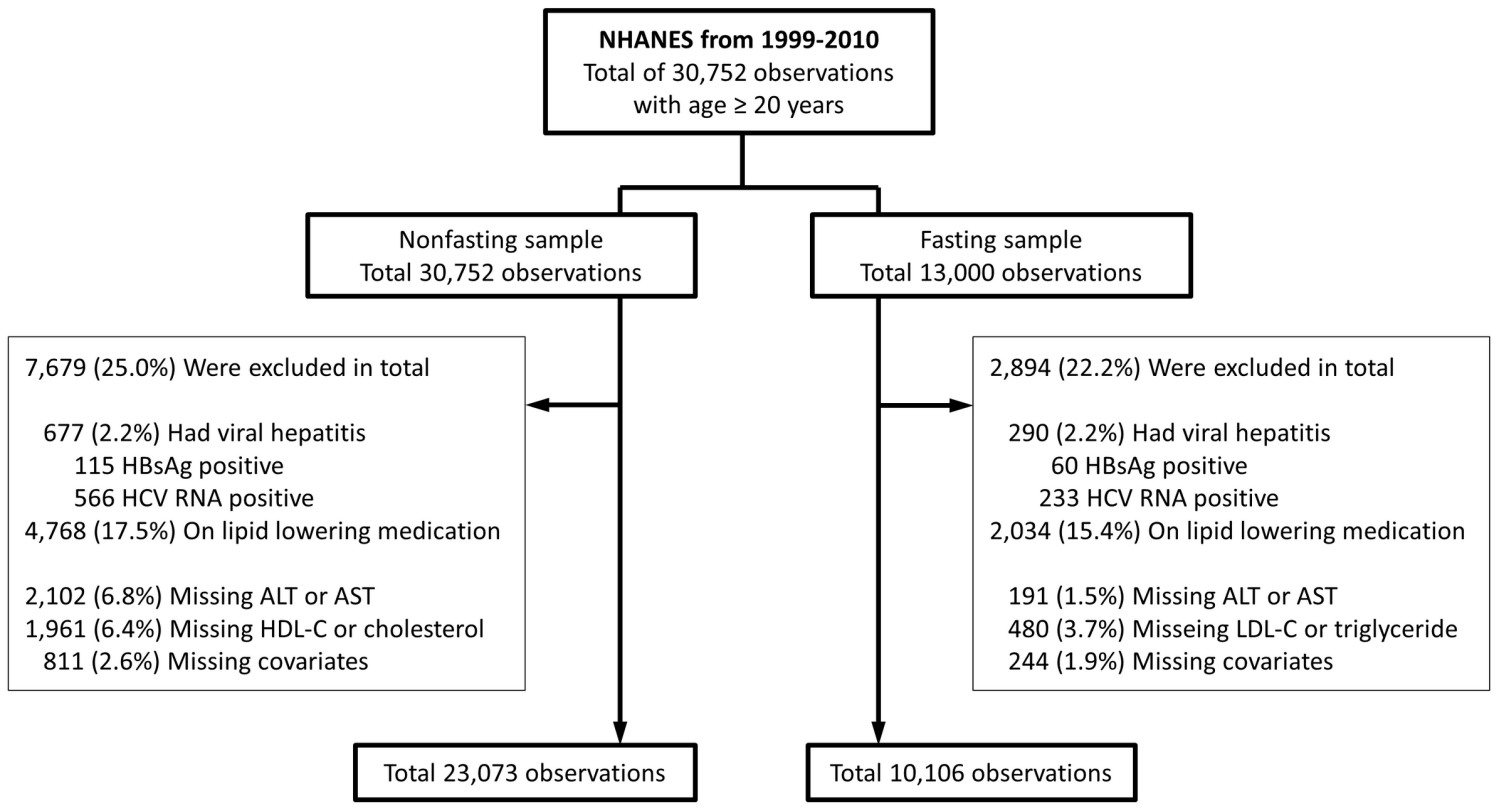
Description of eligible study participants. A total of 30,752 individuals aged 20 years or older were identified from NHANES from 1999 to 2010. Two separate datasets were generated for fasting and nonfasting lab values. In each dataset, participants with evidence of viral hepatitis B or C, currently taking lipid lowering medications, or missing lipoprotein, transaminase, or covariate measurements were excluded. This resulted in a nonfasting dataset of 23,073 observations and fasting dataset of 10,106 observations.

We extracted data on 30,752 individuals with age equal or above 20 years old, who participated in NHANES from 1999 through 2010 (Figure 1). Because not all individuals were fasting at examinations and their LDL-C and triglyceride were not measured, the sample composition and weights differed modestly between LDL-C/triglyceride and total cholesterol/HDL-C. We excluded individuals with a positive hepatitis B surface antigen or positive hepatitis C RNA (n = 677), and those taking lipid lowering medications (n = 4,768), defined by self-reported use of medication for high cholesterol or the presence of a lipid lowering medication on a separate prescription medication inventory. We also excluded participants with missing transaminase levels (n = 2,102), or those with missing data on HDL-C/cholesterol (n = 1,961), or LDL-C/triglyceride (n = 480), or potential confounders (smoking 32, bmi 755, daily number of medications 43, total n = 811), leaving final sample sizes of 23,073 participants for analyses of HDL-C and non-HDL cholesterol and 10,106 participants for analyses of LDL-C and triglyceride.

### Covariates

We identified potential confounders based upon prior studies [3], [21], [22]. Covariates included age, gender, ethnicity, smoking history, alcohol consumption, number of daily medications, and body mass index (BMI). Race and ethnicity were self-reported. Alcohol consumption was self-reported but correlates to the expected degree with HDL-C in previous reports [23]. Alcohol consumption was converted to categorical variables taking into account its non-linear relationship with the outcomes (i.e. AST and ALT levels). Individuals missing alcohol consumption data were included as a separate category in regression models. The number of daily medications was included as a surrogate marker for overall health and to account for unrecognized drug-related hepatotoxicity. Technicians directly measured height and weight, from which BMI was calculated.

### Statistical Analysis

We first examined the continuous association between lipid levels and ALT or AST without assuming linearity by fitting unadjusted restricted cubic spline regression models, with knots corresponding to clinical cutoff points of interest: for LDL-C, 40, 70, 100, 130, 160 mg/dL; for HDL-C, 20, 30, 40, 60, 80, 100 mg/dL; for triglyceride levels, 50, 100, 150, 200, 250 mg/dL; and for non-HDL cholesterol levels, 60, 80, 140, 200, 260 mg/dL. We then constructed multivariate logistic regression models to evaluate the associations of individual lipoprotein lipid classes as well as combined lipid classes with prevalence of abnormal ALT or AST, defined as values greater than 40 IU/L. We chose this cutoff value as it is a common institutional reference value and thus reflects its actual use in the clinical practice. Similar cutoffs have been used in both adolescent and adult epidemiological studies [22], [24]–[26]. We also used a gender-specific cutoff for ALT (male >47 IU/L and female >30 IU/L), and AST greater than 33 IU/L, a reference value recommended in NHANES, as a sensitivity analysis [27]. Each regression model was adjusted for age, age-squared, gender, ethnicity (white, black, Hispanic, other), the number of medications used per day, smoking (never, former, current), alcohol consumption (never, former, <1, 1 to 7, 8 to 14, >14 drinks per week, and missing) and BMI. For interpretability, we treated lipid values as categories using clinical cutoff values: LDL-C ≤40, 41–70, 71–100, 101–130, 131–160, >160 mg/dL; HDL-C ≤30, 31–40, 41–60, 61–80, 81–100, >100 mg/dL; triglyceride ≤50, 51–100, 101–150, 151–200, 201–250, >250 mg/dL, non-HDL cholesterol ≤60, 61–80, 81–140, 141–200, 201–260, >260 mg/dL. The cutoff points were chosen based on the values used in the ATP-III guideline [28], while also accounting for the distribution of values within these samples. In general, categories with the lowest liver enzyme levels were used as the reference levels for the analyses.

To test overlap between lipid types, we simultaneously included the same LDL-C, HDL-C and triglyceride categories using fasting weights, and HDL-C and non-HDL cholesterol categories using non-fasting weights, in single regression models. Given the concern of residual confounding from alcohol consumption, we repeated our analyses among people who drank minimally (<7 drinks per week) as a sensitivity test. We also investigated the impact of gender on our findings through a subgroup analysis using an interaction term between gender and our outcomes of interest (i.e. AST and ALT levels). To test the impact of chronic liver disease, two approaches were taken. We first repeated our analyses following exclusion of individuals who endorsed either active liver disease or a history of liver disease by questionnaire, which resulted in 22,491 non-fasting samples, and 9,847 fasting samples. A second sensitivity test was performed by excluding those with a Fibrosis 4 Score >2.67, indicative of stage 3, 4 fibrosis for NASH, which resulted in 22,459 non-fasting samples, and 9,829 fasting samples. The Fibrosis 4 Score was calculated as [Age(years) × AST(IU/L)]/{Platelet count (×10^9^/L) × [ALT(IU/L))^1/2^]} [29].

All analyses were performed using STATA/IC version 11.0 (Stata Corp, College Station, Texas), accounting for the complex survey design of the study. Taylor series linearization was used for variance estimation [30].

## Results

### Study Participants

Table 1 presents the baseline characteristics of individuals with low, medium or high LDL-C, HDL-C or triglyceride. As expected, LDL-C and triglyceride were positively associated with each other and negatively associated with HDL-C.

**Table 1:** Demographic, clinical and laboratory data of study participants.

	LDL-C, mg/dL				HDL-C, mg/dL				Triglyceride, mg/dL			
	0–70	71–130	>130	p-value*	0–40	41–80	>80	p-value*	0–100	101–200	>200	p-value*
Sample size, N	653	5804	3649		5052	16540	1481		4360	4345	1401	
Age, yr	39±19	42±23	48±21	<0.001	42±20	44±29	49±22	<0.001	41±25	46±22	47±19	<0.001
Gender, %				<0.001				<0.001				<0.001
Male	41.5%	45.0%	50.5%		70.9%	42.3%	19.0%		41.2%	49.4.1%	57.4%	
Race, %				0.003				<0.001				<0.001
White	64.9%	69.0%	71.8%		70.1%	69.5%	74.9%		67.7%	70.8%	73.4%	
Black	16.2%	11.3%	10.1%		7.7%	11.3%	14.2%		15.4%	8.4%	5.2%	
Hispanic	12.8%	14.1%	13.5%		16.6%	13.7%	6.4%		12.0%	15.0%	16.4%	
Smoking status, %				0.02				<0.001				<0.001
Non-smoker	55.7%	54.7%	51.1%		46.8%	55.2%	55.3%		58.1%	50.9%	45.9%	
Former smoker	20.6%	22.5%	25.6%		21.5%	22.9%	26.3%		20.8%	25.1%	27.8%	
Current smoker	23.7%	22.8%	23.3%		31.7%	21.9%	18.4%		21.2%	24.0%	26.3%	
Alcohol drinks per week	3.9±13	4.0±65	3.3±9	0.4	2.9±10	3.7±41	5.6±12	<0.001	3.2±9	4.3±74	3.9±15	0.3
Number of medications	1.6±3.0	1.3±2.9	1.3±2.4	0.03	1.3±3.0	1.3±3.5	1.6±2.7	0.001	1.0±2.3	1.5±2.8	1.7±3.0	<0.001
BMI, Kg/m^2^	27±8	28±9	29±8	<0.001	31±9	28±11	24±6	<0.001	26±8	29±9	31±8	<0.001
Waist circumference, cm	91±22	95±24	98±19	<0.001	104±23	94±29	86±16	<0.001	91±21	99±22	104±19	<0.001
Hypertension, %	24.0%	21.6%	25.5%	0.003	25.6%	21.8%	22.1%	<0.001	16.3%	27.6%	32.5%	<0.001
Hyperlipidemia, %	3.2%	9.9%	32.6%	<0.001	19.2%	17.4%	17.7%	0.06	10.8%	21.1%	29.4%	<0.001
Diabetes, %	6.5%	5.0%	3.9%	0.01	6.7%	4.5%	2.2%	<0.001	2.9%	5.5%	8.6%	<0.001
Coronary artery disease, %	4.0%	1.2%	0.9%	<0.001	1.9%	1.3%	1.2%	0.005	0.8%	1.5%	1.7%	0.08
Stroke, %	3.2%	1.3%	1.8%	0.001	2.2%	1.6%	1.7%	0.04	1.2%	1.9%	1.7%	0.08
Cancer, %	7.5%	6.8%	7.9%	0.2	5.2%	7.4%	10.6%	<0.001	6.1%	8.2%	7.7%	<0.001
history of liver disease, %	2.5%	2.3%	2.9%	0.3	2.9%	2.3%	2.3%	0.1	1.7%	2.4%	3.8%	0.009
Active liver disease, %	1.8%	0.8%	0.9%	0.09	1.2%	0.8%	0.6%	0.08	0.6%	0.8%	1.4%	0.2
LDL-C (mg/dL)	60±11	104±20	157±32	<0.001	121±70	121±74	111±61	<0.001	110±39	129±50	127±48	<0.001
HDL-C (mg/dL)	59±23	55±24	52±19	<0.001	35±6	56±16	92±14	<0.001	60±23	51±21	43±15	<0.001
Triglyceride (mg/dL)	105±87	116±79	140±85	<0.001	215±396	119±154	85±62	<0.001	72±24	140±34	259±60	<0.001
ALT (U/L)	22±17	24±17	28±55	<0.001	32±49	24±70	22±18	<0.001	22±16	26±23	33±87	<0.001
AST (U/L)	24±13	24±16	26±21	<0.001	26±19	24±18	27±19	<0.001	24±17	25±22	26±17	0.003

P-values were calclulated using ANOVA for continous variables and chi-square test for categorical variables.

### U shaped Associations between ALT/AST and LDL-C/HDL-C

**Figure 2:**
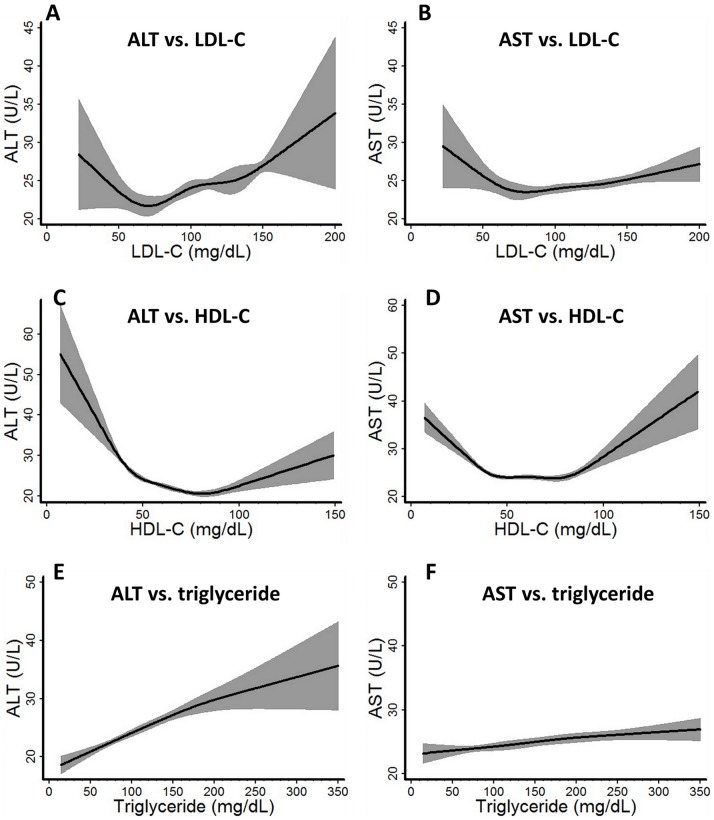
Association curves between ALT, AST and LDL-C, HDL-C and triglyceride. The relationship between ALT, AST and LDL-C, HDL-C and triglycerides were modeled with unadjusted restricted cubic spline models. Evenly distributed conventional lipid profile cutoff points were chosen as knots in generating the model, with LDL-C at 40, 70, 100, 130, 160 mg/dL; HDL-C at 20, 30, 40, 60, 80, 100 mg/dL, and triglyceride at 50, 100, 150, 200, 250 mg/dL. Sample weights were taken into consideration during the modeling to represent the association in the general US population.

We first explored the shape of the relationship between serum lipid profile and transaminase levels using restricted cubic splines with knots evenly set at clinically determined cutoff points. Of note, unlike most regression models, restricted cubic spline does not assume a predetermined shape of the association curve, but rather allow the data to determine its shape. Both ALT and AST demonstrated a non-linear U-shaped association with LDL-C and HDL-C, but not with triglyceride (Figure 2). LDL-C and triglyceride generally had stronger associations with ALT than AST, while that for HDL-C was similar for both ALT and AST. We also tested the relationships between ALT, AST, and total, non-HDL cholesterol, which used the nonfasting dataset. These showed similar U shaped associations with transaminase levels as did LDL-C (data not shown).

### Low LDL-C and High HDL-C are Associated with Abnormal Liver Enzymes

To further characterize this association, we calculated the odds ratio of abnormal ALT (ALT >40 IU/L), AST (AST >40 IU/L) or either liver enzyme (ALT or AST >40 IU/L) using logistic regression models (Table 2). After adjustment, high LDL-C was associated with higher odds of elevated transaminases. LDL-C less than 40 mg/dL was associated with four-fold higher odds for abnormal ALT, seven-fold for abnormal AST, and four-fold for any abnormal liver enzyme, compared to those with LDL-C between 71–100 mg/dL. Half a million (95% CI 0.4–0.7 million) US citizens, or 0.3% of the tested US adult population (0.2% of the total US population) have an LDL less than 40 mg/dL, and approximately one in five individuals in this group have an abnormal transaminase (Table 3). Similarly, an LDL-C between 41 and 70 mg/dL, considered a target LDL range for many individuals, was associated with an odds ratio of 1.6 for abnormal liver enzymes compared to the reference group (Table 2). Approximately 9.5 million (95% CI 8.4–10.6 million) US citizens have an LDL-C in this range, representing at least 5.7% of the tested US adult population (3.6% of the total US population) (Table 3).

**Table 2:** Adjusted association between lipid profile and the probability of elevated liver enzymes.

		ALT >40 U/L			AST >40 U/L			ALT >40 U/L or AST >40 U/L		
	N	OR	95% CI	p	OR	95% CI	P	OR	95% CI	p
**LDL-C, mg/dL** (n = 10106)										
0–40	39	**3.8** *	**1.2–12.3**	**0.03**	**6.9**	**2.2–21.2**	**0.001**	**4.2**	**1.5–11.7**	**0.007**
41–70	614	1.5	0.9–2.3	0.1	1.7	0.9–3.0	0.08	**1.6**	**1.1–2.5**	**0.03**
71–100	2368	1.0	Ref.	Ref.	1.0	Ref.	Ref.	1.0	Ref.	Ref.
101–130	3436	1.2	0.90–1.6	0.2	1.1	0.8–1.7	0.5	1.2	0.9–1.6	0.23
131–160	2380	**1.8**	**1.4–1.6**	**<0.001**	1.4	1.0–2.2	0.08	**1.7**	**1.3–2.1**	**<0.001**
>160	1269	**2.1**	**1.6–2.7**	**<0.001**	**1.7**	**1.1–2.5**	**0.02**	**2.0**	**1.5–2.6**	**<0.001**
**HDL-C, mg/dL** (n = 23073)										
0–30	887	**3.4**	**2.5–4.7**	**<0.001**	**2.2**	**1.4–3.3**	**<0.001**	**2.7**	**2.0–3.6**	**<0.001**
30–40	4165	**2.2**	**1.8–2.8**	**<0.001**	1.3	0.9–1.7	0.1	**1.8**	**1.5–2.2**	**<0.001**
41–60	11474	**1.4**	**1.2–1.7**	**<0.001**	0.8	0.7–1.0	0.07	1.1	1.0–1.3	0.1
61–80	5066	1.0	Ref.	Ref.	1.0	Ref.	Ref.	1.0	Ref.	Ref.
81–100	1206	1.0	0.6–1.6	0.99	1.5	1.0–2.3	0.09	1.2	0.8–1.7	0.3
>100	275	**2.3**	**1.3–4.0**	**0.01**	**4.4**	**2.8–7.0**	**<0.001**	**3.2**	**2.1–5.0**	**<0.001**
**Triglyceride, mg/dL** (n = 10106)										
0–50	567	0.7	0.4–1.3	0.2	**2.9**	**1.7–5.0**	**0.04**	1.4	0.8–2.3	0.2
51–100	3793	1.0	Ref.	Ref.	1.0	Ref.	Ref.	1.0	Ref.	Ref.
101–150	2879	**1.7**	**1.3–2.1**	**<0.001**	**1.4**	**1.0–1.9**	**0.06**	**1.6**	**1.3–2.0**	**<0.001**
151–200	1466	**1.9**	**1.4–2.5**	**<0.001**	1.3	0.9–1.7	0.1	**1.8**	**1.3–2.3**	**<0.001**
201–250	744	**2.6**	**1.9–3.4**	**<0.001**	**2.0**	**1.4–3.0**	**0.001**	**2.4**	**1.8–3.2**	**<0.001**
>250	657	**2.4**	**1.7–3.4**	**<0.001**	**2.1**	**1.3–3.4**	**0.003**	**2.3**	**1.6–3.1**	**<0.001**

odds ratios with p value <0.05 are highlighted in bold.

**Table 3:** Prevalence of elevated transaminases at different LDL-C and HDL-C levels.

	Population, million(% eligible population)	95% CI,million	Population with abnormalALT or AST, million	95% CI,million	% lipidsubgroup
**LDL-C**					
≤40	0.5 (0.3%)	0.4–0.7	0.1	0.0–0.2	20.6%
41–70	9.5 (5.7%)	8.4–10.6	0.9	0.6–1.3	10.0%
71–100	40.4 (24.3%)	38.1–42.8	3.1	2.5–3.6	7.6%
101–130	57.1 (34.4%)	53.3–60.9	5.2	4.4–6.0	9.2%
131–160	38.2 (23.0%)	35.3–41.1	4.7	4.1–5.4	12.4%
>160	19.9 (12.0%)	18.2–21.6	2.7	2.1–3.3	13.4%
**Subtotal**	**166.0 (100%)**	**157.0–174.0**	**16.8**	**15.3–18.3**	**10.4%**
**HDL-C**					
≤30	5.0 (3.1%)	4.4–5.5	1.4	1.1–1.6	27.1%
31–40	29.8 (18.4%)	27.7–31.9	5.4	4.8–6.0	18.2%
41–60	80.0 (49.4%)	76.3–83.7	7.4	6.8–7.9	9.2%
61–80	35.2 (21.7%)	33.4–37.1	2.0	1.7–2.3	5.8%
81–100	8.3 (5.1%)	7.6–9.0	0.5	0.3–0.6	5.6%
>100	1.8 (1.1%)	1.5–2.1	0.2	0.1–0.3	11.9%
**Subtotal**	**162.0 (100%)**	**155.0–169.0**	**17.2**	**16.1–18.2**	**10.6%**

Low HDL-C was associated with an abnormal ALT or AST, as expected. However, elevated HDL-C above 100 mg/dL was also associated with a two-fold increase in odds ratios for abnormal ALT, four-fold increase for abnormal AST and three-fold increase for either abnormal ALT or AST compared to the group with HDL-C between 61 and 80 mg/dL (Table 2). Approximately 1.8 million (95% CI 1.5–2.1 million) US citizens, or 1.1% of the tested US adult population, have levels of HDL-C above 100 mg/dL, and approximately 11.9% of this tested population have elevated liver enzymes (Table 3).

Because residual confounding by alcohol consumption could potentially affect the shape of the association between lipid profile and liver functions, we repeated our analyses after excluding former or current drinkers who reported more than 7 drinks per week. This did not affect our estimates of the odds ratio for abnormal ALT (adjusted odds ratio 2.4; 95% CI 1.1–5.4, p = 0.04), but the odds ratio for abnormal AST was attenuated to 2.7 (95% CI 1.4–5.6, p = 0.006), indicating that residual confounding may contribute to our HDL-C findings, but is unlikely to account for this observation entirely.

Because males and females differ in both serum transaminases and lipid profiles [31], we performed sub-group analyses comparing the associations between males and females. In general, the odds ratios in males tended to be larger than those in females at both low LDL-C (LDL-C ≤40, 41–70 mg/dL) and high HDL-C (HDL-C >100 mg/dL), but none of the gender differences reached statistical significance in formal tests of interaction (data not shown).

Similarly, female transaminase levels are generally lower than males [31], so we performed a sensitivity analysis using a gender-specific ALT cutoff (47 U/L for male, 30 U/L for female) and AST cutoff of 33 U/L, the thresholds NHANES recommends [27]. An LDL-C less than 40 mg/dL was associated with an odds ratio of 3.3 (95% CI 1.9–5.8, p<0.001) for abnormal liver enzymes, although LDL-C between 41 and 70 mg/dL was not (adjusted odds ratio 1.2; 95% CI 0.8–1.8, p = 0.3). When using these cutoff values for HDL-C, not only was HDL-C more than 100 mg/dL associated with a 3.1 fold increase (95% CI 2.1–4.4, p<0.001) in the odds of abnormal liver enzymes, but HDL-C between 80–100 mg/dL also showed statistically significant association with a 1.4 fold increase (95% CI 1.1–1.8, p = 0.005) in the odds of abnormal liver enzymes.

To test the possibility that the association between abnormal liver enzymes and low LDL-C or high HDL-C is largely driven by known liver disease, we performed exploratory analyses excluding individuals with either current or former self-reported liver disease. No substantial differences were found. LDL-C ≤40 and 41–70 mg/dL had a 3.4 (95% CI 1.1–10.6, p = 0.03) and 1.6 (95% CI 1.0–2.5, p = 0.04) fold increases in odds of abnormal liver enzymes respectively, while HDL-C >100 mg/dL had a 3.3 (95% CI 2.2–4.8, p<0.001) fold increase in odds of abnormal liver enzymes (Table S1). The exclusion of those with FIB4 score >2.67 resulted in a very similar attenuation in the odds ratio and decrease in p value at low LDL-C or high HDL-C, but on significant impact on the overall association was observed. The odds ratio of abnormal LFT for LDL-C ≤40 and 41–70 mg/dL were 3.0 (95% CI 0.9–10.1, p = 0.07) and 1.7 (95% CI 1.1–2.7, p = 0.02) respectively compared to LDL-C 71–100 mg/dL, whereas the odds ratio for HDL-C >100 mg/dL was 3.2 (95% CI 1.5–7.0, p = 0.003) compared to HDL-C 61–80 mg/dL (Table S2). In contrast to the relationships with LDL-C and HDL-C, the relationship between triglyceride and ALT was approximately linear (Table 2). To evaluate for the presence of independent associations, we modeled the odds of abnormal ALT or AST using LDL-C, HDL-C, triglyceride, and other covariates simultaneously. The odds ratios were 4.1 (95% CI 1.5–11.7, p = 0.008) at LDL-C of 0–40 mg/dL, 1.6 (95% CI 1.0–2.5, p = 0.04) at LDL-C of 41–70 mg/dL, and 4.2 (95% CI 2.4–7.4, p<0.001) at HDL-C >100 mg/dL. These findings were similar to the odds ratios calculated using individual lipid types (Table 2). In comparison, a small but consistent decrease in the odds ratios for low HDL-C and high triglyceride were observed when all three lipid groups were used, suggesting an overlap of their effect on liver enzymes in these lipid ranges (data not shown).

## Discussion

This study represents a comprehensive attempt to examine the relationship between serum lipid profiles and serum transaminase levels. Both low LDL-C and high HDL-C values, an often perceived as a desirable lipid panel, were paradoxically associated with significantly higher prevalence of abnormal levels of ALT and AST. Accordingly, some 10 million American adults with an LDL-C less than 70 mg/dL, and 1.8 million with HDL-C more than 100 mg/dL are at increased risk for potentially unrecognized liver injury. As we excluded people with viral hepatitis and those taking lipid lowering medications, metabolic liver disease is the most likely cause of these injuries.

Despite the routine and widespread use of serum lipid panels, the utility in the assessment of known or unknown liver diseases has been underappreciated for two main reasons. First, the fasting lipoproteins assayed by the lipid panel – mainly apoA1- and apoB-containing lipoproteins – are essentially all produced by the liver [2]. Hepatocytes dictate the secretion of VLDL, which is later converted to LDL in the circulation. These apoB-containing lipoproteins account for almost all serum triglycerides and majority of serum cholesterol. Secondly, hepatocytes also actively uptake circulating LDL-C and HDL-C via LDL receptors (LDLR) and scavenger receptors (SR-BI), which in turn fill the intrahepatic lipid pool and deplete the circulating lipid pool. Lipid homeostasis in the liver thus exerts a profound effect on measured serum LDL-C, HDL-C, and triglycerides [1], [32], [33].

The causes of elevated liver transaminase levels among individuals with ostensibly “optimal” lipid profiles are likely multifactorial, and may differ between LDL-C and HDL-C. Cross-sectional design of the study did not eliminate the possibility of reverse causality. In fact, the physiology of hepatic lipoprotein metabolism indicates a potentially bidirectional relationship. For LDL-C, disorders of lipoprotein metabolism can lead to hepatic injury, whereas chronic liver disease may also impair lipoprotein production.

FHBL and abetalipoproteinemia are two well-established causes of hepatic steatosis and elevated transaminases. They are generally considered rare entities. FHBL has an estimated prevalence of 1/500 to 1/1000, similar to type 1 diabetes, while abetalipoproteinemia is even rarer [13], [34], [35]. The prevalence that we observed here far exceeds the known incidence of these two conditions, suggesting alternative causes or an underestimation of these conditions. The sheer size and hydrophobic nature of VLDL and its complicated path from the endoplasmic reticulum to excretion requires an orchestrated assembly of cellular components, each subject to genetic alternations. Genome wide association studies have identified at least 95 genetic loci that can potentially influence serum lipoprotein profiles, testifying to the complex nature of this process [36].

Secondly, chronic liver disease can lead to acquired hypobetalipoproteinemia. Indeed, cirrhosis is a known state with low VLDL production rate secondary to the loss of liver synthetic function. It has been reported that apoB synthesis is impaired in NASH compared to BMI matched obese controls [37]. Meanwhile, progressive insulin resistance has been linked to significantly reduced microsomal triglyceride transfer protein (MTP) expression, a protein that facilitates apoB maturation, thus impairing VLDL secretion [38]. The fact that our results attenuated a little with exclusion of individuals who reported known liver disease or had biomarkers suggestive of advanced fibrosis suggests that underlying liver disease or cirrhosis is indeed a potential cause contributing to this association. It is interesting that individuals in the lowest LDL-C group had the highest rate of coronary artery disease and stroke (Table 1). This might be influenced by a higher rate of insulin resistance and diabetes, leading to both NASH cirrhosis and vascular diseases.

We found an unexpected association between elevated liver enzymes and elevated HDL-C, also known as hyperalphalipoproteinemia (HALP). In contrast with hypobetalipoproteinemia, we observed more pronounced AST elevations in HALP, suggesting different underlying mechanisms. Known genes that lead to HALP include cholesteryl ester transfer protein (CETP), hepatic lipase and endothelial lipase, but their hepatic manifestations are not well characterized ^25, 32^. Alcohol is a potential contributor to this association, as it increases both the HDL-C and liver enzymes, especially AST [39]. While we adjusted for alcohol in our analyses, residual confounding from underreporting of alcohol use could contribute to this association. Most intriguingly, the elevated HDL-C could also be a direct result of hepatic injury. It is increasingly evident that HDL functions as more than a lipid carrier and plays important roles in inflammation, thrombosis, and endothelial integrity [40]. It carries a host of apolipoproteins along with complement regulatory proteins and inhibitors for endopeptidase [41]. It is unclear whether both elevated liver enzymes and HDL-C are results of more systemic processes.

The large sample size and the generalizability to the US population are strengths of this study. The high quality of NHANES survey provides extensive and reliable information on the status of viral hepatitis, the use of lipid-lowering medication, and alcohol and smoking history [20]. However, our study has several limitations. First, we used transaminases as a surrogate marker, which is an indirect assessment of liver diseases. NAFLD is likely to be the predominant etiology for the observed abnormalities, but without actual liver fat measurement, heterogeneity in liver pathology should be presumed. Second, the proportions of people with low LDL-C and high HDL-C are small. Therefore, despite the large dataset, the power can be limited in these categories, especially when the sample size is reduced for sensitivity analysis. Third, only a single measurement of transaminase levels was available for each individual in the NHANES data. Therefore, abnormal liver function tests in our study indicated the existence of chronic liver disease, but they were not equivalent to a diagnosis of chronic liver disease, which by definition requires at least two sets of abnormal liver function test over a six months period. Finally, we intentionally excluded individuals taking lipid lowering medications. Our findings should not be extrapolated to population with hyperlipidemia and on lipid modifying medications. Current ATP III guidelines recommend a treatment goal of LDL less than 70 among individuals with coronary artery disease or its equivalent [28]. Our study should not be interpreted as evidence for hepatic side effects for this treatment goal, as none of our study sample had their serum lipid profile decreased by pharmacological means.

In summary, both low LDL-C and high HDL-C were associated with significantly higher odds of elevated liver enzymes in the general U.S. adult population. Our findings raise concerns about potentially unrecognized hepatic dysfunction among people with particularly low LDL-C or high HDL-C. The underlying hepatic pathophysiology deserves further exploration.

## Supporting Information

Table S1 **Association between abnormal ALT, AST and LDL-C, HDL-C among those without self-reported liver disease.**
(DOCX)

Table S2 **Association between abnormal ALT, AST and LDL-C, HDL-C among those without stage 3, 4 fibrosis by FIB4 score.**
(DOCX)
