# Supplementary material for: Low LDL-C and High HDL-C Levels Are Associated with Elevated Serum Transaminases amongst Adults in the United States: A Cross-sectional Study
Source: PLoS One. 2014 Jan 15;9(1):e85366. doi: 10.1371/journal.pone.0085366 (PMC3893181; doi:10.1371/journal.pone.0085366)
Supplement: Table S1 — Association between abnormal ALT, AST and LDL-C, HDL-C among those without self-reported liver disease. (DOCX) [file pone.0085366.s001.docx]

**Table S1 Association between abnormal ALT, AST and LDL-C, HDL-C among those without self-reported liver disease**

|  |  | **ALT > 40 U/L** | | | **AST > 40 U/L** | | | **ALT > 40 or AST > 40 U/L** | | |
| --- | --- | --- | --- | --- | --- | --- | --- | --- | --- | --- |
|  | N | OR | 95% CI | p-value | OR | 95% CI | p-value | OR | 95% CI | p-value |
| **LDL-C (mg/dL)** | |  |  |  |  |  |  |  |  |  |
| 0 – 40 | 37 | 3.2 | 0.9 - 11.5 | 0.07 | 5.9 | 1.7 - 20.5 | 0.006 | **3.4** | **1.1 - 10.6** | **0.03 ^1^** |
| 41 – 70 | 603 | 1.4 | 0.9 - 2.2 | 0.15 | 1.6 | 0.9 - 3.0 | 0.1 | **1.6** | **1.0 - 2.5** | **0.04** |
| 71 – 100 | 2317 | 1.0 | REF | REF | 1.0 | REF | REF | 1.0 | REF | REF |
| 101 – 130 | 3349 | 1.3 | 0.9 - 1.7 | 0.12 | 1.2 | 0.8 - 1.8 | 0.4 | 1.3 | 0.9 - 1.7 | 0.1 |
| 131 – 160 | 2307 | **1.9** | **1.4 - 2.4** | **<0.001** | **1.6** | **1.0 - 2.4** | **0.04** | **1.7** | **1.3 - 2.2** | **<0.001** |
| > 160 | 1234 | **2.1** | **1.5 - 2.8** | **<0.001** | **1.6** | **1.1 - 2.6** | **0.03** | **2.0** | **1.5 - 2.7** | **<0.001** |
|  |  |  |  |  |  |  |  |  |  |  |
| **HDL-C (mg/dL)** | |  |  |  |  |  |  |  |  |  |
| 0 – 30 | 854 | **3.3** | **2.4 - 4.5** | **<0.001** | **2.0** | **1.3 - 3.1** | **0.002** | **2.6** | **2.0 - 3.5** | **<0.001** |
| 31 – 40 | 4037 | **2.2** | **1.8 - 2.7** | **<0.001** | 1.2 | 0.9 - 1.7 | 0.1 | **1.8** | **1.5 - 2.2** | **<0.001** |
| 41 – 60 | 11186 | **1.4** | **1.2 - 1.6** | **<0.001** | **0.8** | **0.6 - 1.0** | **0.02** | 1.1 | 1.0 - 1.3 | 0.2 |
| 61 - 80 | 4962 | 1.0 | REF | REF | 1.0 | REF | REF | 1.0 | REF | REF |
| 81 – 100 | 1183 | 1.0 | 0.6 - 1.7 | 0.9 | 1.5 | 0.9 - 2.4 | 0.09 | 1.2 | 0.8 - 1.8 | 0.3 |
| > 100 | 269 | **2.2** | **1.2 - 3.9** | **0.009** | **4.4** | **2.9 - 6.8** | **<0.001** | **3.3** | **2.2 - 4.8** | **<0.001** |

^1^ P value ≤ 0.05 highlighted in bold
