# Supplementary material for: Low LDL-C and High HDL-C Levels Are Associated with Elevated Serum Transaminases amongst Adults in the United States: A Cross-sectional Study
Source: PLoS One. 2014 Jan 15;9(1):e85366. doi: 10.1371/journal.pone.0085366 (PMC3893181; doi:10.1371/journal.pone.0085366)
Supplement: Table S2 — Association between abnormal ALT, AST and LDL-C, HDL-C among those without stage 3, 4 fibrosis by FIB4 score. (DOCX) [file pone.0085366.s002.docx]

**Table S2 Association between abnormal ALT, AST and LDL-C, HDL-C among those without stage 3, 4 fibrosis by FIB4 score**

|  |  | **ALT > 40 U/L** | | | **AST > 40 U/L** | | | **ALT > 40 or AST > 40 U/L** | | |
| --- | --- | --- | --- | --- | --- | --- | --- | --- | --- | --- |
|  | N | OR | 95% CI | p-value | OR | 95% CI | p-value | OR | 95% CI | p-value |
| **LDL-C (mg/dL)** | |  |  |  |  |  |  |  |  |  |
| 0 – 40 | 33 | **3.4** | **1.0 - 11.6** | **0.05 ^1^** | **5.4** | **1.3 - 22.9** | **0.02** | 3.0 | 0.9 - 10.1 | 0.07 |
| 41 – 70 | 579 | 1.5 | 0.9 - 2.4 | 0.1 | 1.8 | 1.0 - 3.5 | 0.07 | **1.7** | **1.1 - 2.7** | **0.02** |
| 71 – 100 | 2280 | 1.0 | REF | REF | 1.0 | REF | REF | 1.0 | REF | REF |
| 101 - 130 | 3359 | 1.3 | 0.9 - 1.7 | 0.1 | 1.3 | 0.9 - 2.0 | 0.2 | 1.3 | 0.9 - 1.7 | 0.1 |
| 131 – 160 | 2332 | **1.9** | **1.5 - 2.4** | **< 0.001** | **1.8** | **1.2 - 2.7** | **0.008** | **1.8** | **1.4 - 2.4** | **< 0.001** |
| > 160 | 1246 | **2.2** | **1.7 - 2.9** | **< 0.001** | **2.1** | **1.4 - 3.2** | **< 0.001** | **2.2** | **1.6 - 2.9** | **< 0.001** |
|  |  |  |  |  |  |  |  |  |  |  |
| **HDL-C (mg/dL)** | |  |  |  |  |  |  |  |  |  |
| 0 - 30 | 857 | **3.1** | **1.9 - 5.1** | **< 0.001** | **2.1** | **1.1 - 4.1** | **0.03** | **2.7** | **1.7 - 4.4** | **< 0.001** |
| 31 – 40 | 4060 | **2.3** | **1.7 - 3.2** | **< 0.001** | 1.4 | 0.9 - 2.3 | 0.1 | **2.0** | **1.5 - 2.7** | **< 0.001** |
| 41 - 60 | 11221 | **1.5** | **1.1 - 1.9** | **0.005** | 1.1 | 0.8 - 1.6 | 0.5 | 1.3 | 1.0 - 1.6 | 0.06 |
| 61 - 80 | 4912 | 1.0 | REF | REF | 1.0 | REF | REF | 1.0 | REF | REF |
| 81 - 100 | 1157 | 1.0 | 0.5 - 1.9 | 1.0 | 1.5 | 0.6 - 3.4 | 0.4 | 1.3 | 0.7 - 2.4 | 0.4 |
| > 100 | 252 | 2.1 | 0.9 - 4.6 | 0.07 | **6.5** | **2.9 - 14.7** | **< 0.001** | **3.2** | **1.5 - 7.0** | **0.003** |

^1^ P value ≤ 0.05 highlighted in bold
